# A structure-based gamma evaluation method for identifying clinically relevant dose differences in organs at risk

**DOI:** 10.1007/s13246-023-01270-3

**Published:** 2023-05-23

**Authors:** Liting Yu, Anthony Baker, Tanya Kairn, Alexander Livingstone, Jamie Trapp, Scott B Crowe

**Affiliations:** 1 Royal Brisbane and Women’s Hospital, Herston, 4029 Australia; 2 Queensland University of Technology, Brisbane, 4001 Australia; 3 Nepean Hospital, Kingswood, 2747 Australia

**Keywords:** Structure-based, Gamma evaluation, Organs-at-risk, Clinically relevant, Patient specific quality assurance, PSQA

## Abstract

Gamma evaluation is currently the most widely used dose comparison method for patient specific quality assurance (PSQA). However, existing methods for normalising the dose difference, using either the dose at the global maximum dose point or at each local point, can respectively lead to under- and over-sensitivity to dose differences in organ-at-risk structures. This may be of concern for plan evaluation from clinical perspectives. This study has explored and proposed a new method called structural gamma, which takes structural dose tolerances into consideration while performing gamma analysis for PSQA. As a demonstration of the structural gamma method, a total of 78 retrospective plans on four treatment sites were re-calculated on an in-house Monte Carlo system and compared with doses calculated from the treatment planning system. Structural gamma evaluations were performed using both QUANTEC dose tolerances and radiation oncologist specified dose tolerances, then compared with conventional global and local gamma evaluations. Results demonstrated that structural gamma evaluation is especially sensitive to errors in structures with restrictive dose constraints. The structural gamma map provides both geometric and dosimetric information on PSQA results, allowing straightforward clinical interpretation. The proposed structure-based gamma method accounts for dose tolerances for specific anatomical structures. This method can provide a clinically useful method to assess and communicate PSQA results, offering radiation oncologists a more intuitive way of examining agreement in surrounding critical normal structures.

## Introduction

Gamma evaluation [1] is currently the most widely used metric for radiotherapy dose distribution comparison [2]. The gamma evaluation is a popular method for analysing patient specific quality assurance (PSQA) results for modulated radiotherapy, including intensity modulated radiation therapy (IMRT) and volumetric modulated arc therapy (VMAT). The gamma evaluation method can also be used for comparing any two dose distributions, for example, comparing measured and calculated dose profiles, or dose distributions from a treatment planning system (TPS) and an independent dose check system.

Gamma evaluation has the advantages of analysing in both dosimetric and spatial domain, and producing an array of gamma values, often represented as a two-dimensional colour map or reported as a single numeric gamma agreement index or percentage pass rate. This comparison that takes into account both dose-differences and distances-to-agreement (DTAs) is especially useful for comparing dose distributions from modulated (IMRT and VMAT) radiotherapy treatments, where inverse planning optimisation can create complex dose distributions that achieve tumoricidal doses to planning target volumes (PTVs) while complying with specified dose constraints for organ-at-risk (OAR) structures.

There are two established methods for calculating the gamma value at each point in the distribution. The global gamma method calculates percentage dose differences with reference to the global maximum dose in the dose distribution, which often approximates the prescription dose [1]. The local gamma method calculates percentage dose differences with reference to the local dose at each point. Although very useful for highlighting dose differences throughout the distribution, the local gamma method has the potential disadvantage of over-emphasising large percentage dose differences at comparatively low dose points [3, 4]. This may lead to over-sensitivity to dose differences in out-of-field regions where these dose differences may be clinically acceptable, depending on the dose constraints in these regions and whether the planned dose is substantially lower than those constraints. The global gamma method may be subject to the opposite issue, ignoring dose differences that are small compared to the global maximum, even when these differences occur in critical organ-at-risk structures [5]. Recently, Baran et al [6] provided a theoretical demonstration that there cannot be any correlation between the standard global gamma index pass rate and common dose-volume metrics.

Given these persistent concerns regarding the clinical relevance of gamma evaluation results, several studies in the literature have explored alternative ways of dose distribution comparisons [5, 7, 8], some groups have attempted to produce clinically relevant comparisons by applying or reporting the gamma evaluation in novel ways [9–11]. For example, van der Bijl et al [12] took advantage of the features of the global gamma method to perform evaluations using only data within the 50% isodose (effectively applying a 50% low dose threshold and considering only the treated volume) which were shown to correlate well with PTV dose volume metrics. Cozzolino et al. and Yi et al [13–15] introduced a concept called volume-based gamma, where gamma analysis results were calculated using the global gamma method, with uniform evaluation criteria, and reported improved correlation with dose-volume metrics.

The potential value of volume-based gamma evaluation methods is growing, as calculation-based PSQA methods are increasingly adopted. Current options include log-based calculations and EPID dose reconstruction [16, 17] as well as comparisons with dose distributions calculated using secondary check systems [18] or independent Monte Carlo (MC) systems [19, 20]. The report of AAPM Task Group 218 [2] (TG-218) explicitly recommended the use of dose difference criterion customised for each organ to allow for clinically relevant criteria to detect clinically relevant errors.

In this study we developed a tool to perform gamma calculation based on individuated structural dose tolerances. We have developed a method that is able to calculate gamma on a structure basis, facilitating the use of a different clinically relevant dose difference criteria for each contoured structure. It is expected that this *structural gamma* method will avoid the issues with both global and local gamma and allow clinicians to interpret PSQA results with reference to specific dose objectives and constraints that are relevant to each irradiated organ.

## Methods

**Fig. 1:**
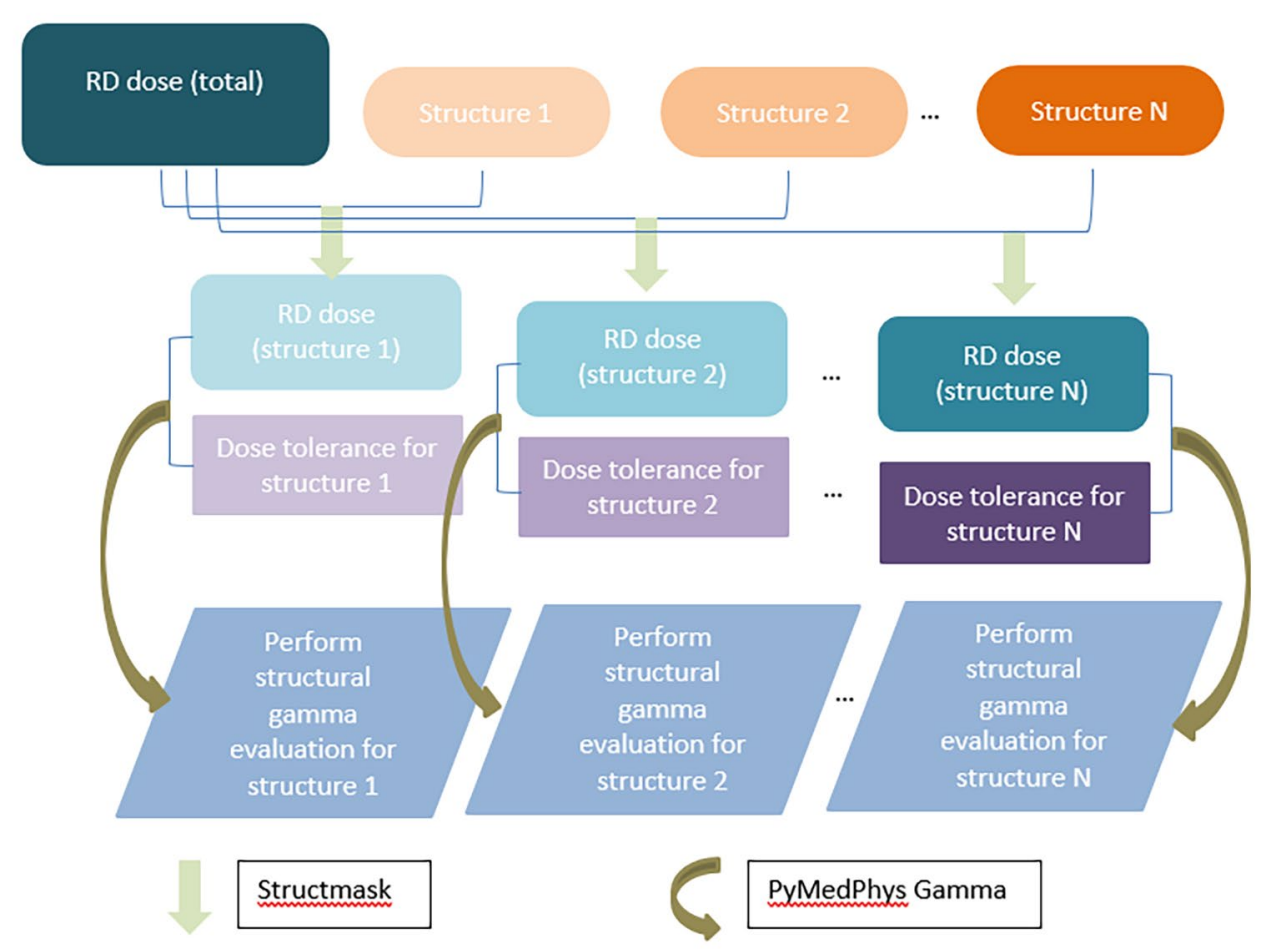
Flowchart of how the structural gamma doses are calculated using *Structmask* and *PyMedPhys* gamma codes

In order to implement the proposed structural gamma method, an in-house Python [21] (version 3.6, Python Software Foundation, Wilmington, USA) code named *Structmask* was developed. The doses from TPS and MC were first calculated and exported to the RTDOSE dataset for the patient plan. *Structmask* creates an RTDOSE dataset for each structure from a given DICOM dose and structure dataset exported from the TPS and MC. The code makes a mask of each structure using a point in polygon method from the matplotlib [22] Python library, then creates a dataset for each structure using this mask where the dose values outside the structure are set to zero. For example, if a head-and-neck treatment plan has 50 contoured structures, *Structmask* will produce 50 structure-masked dose distributions in under one minute. The resulting dose distributions can then be compared using gamma evaluation with criteria specifically selected to apply to each contoured structure. The workflow of this method is illustrated in the flowchart in Fig. 1. Note that the organ specific dose tolerance only applies to the specific organ and is not used for the entire dose distribution. Rather, the structural gamma evaluation iteratively works through each contoured structure, selecting gamma criteria that relate to that structure, and performing the gamma evaluation for that structure only. Points outside the structure in question are not included in the gamma evaluation and do not contribute to the resulting pass rate for that structure. The in-house MC system also looks for contour names such as “bolus” or “artifact” and automatically convert them to water. These structures were later excluded from the gamma evaluation.

To evaluate the structural gamma method, an ethics approved retrospective study of 78 past VMAT plans was completed. These plans were arbitrarily selected from patients treated at our department with standard fractionation between 2019 and 2020. Efforts have been made to minimise the inclusion of palliative or boost plans where the prescription dose was non-standard. The number of treatment plans selected from each of the four anatomical sites are, brain: 20; head-and-neck: 19; thorax: 20; pelvis: 19. More details of the treatment plans analysed are listed in Table 1. All treatments were planned in the Eclipse TPS v13.7 (Varian Medical Systems, Palo Alto, California, USA), using photon optimiser (PO) v13.7.14 and Acuros XB (AXB) v13.7.14 dose calculation algorithm with 2 mm grid size. All plans were exported from the TPS to an in-house MC system and re-calculated, using the methods previously described in the literature [19, 23]. The in-house MC system takes the same dose grid as the TPS and performs its dose calculation. For each plan, the total dose file (RTDOSE, contains the total volumetric dose received by the patient) and the structure file (RTSTRUCT, contains all contoured structures) from both TPS and MC were exported in DICOM format and structure-masked dose distributions were generated using *Structmask*.

Gamma evaluations with four different types of dose difference normalisation were performed for each structure between the structure-masked dose distributions from MC (evaluated dataset) and TPS (reference dataset) using the PyMedPhys [24] v0.35.0 gamma code, with a tolerance of 2%, 2 mm and a low dose threshold of zero. The normalisation values were: global maximum dose, local dose, QUANTEC [25] dose tolerances, and radiation oncologist (RO) specified dose tolerances. The first two dose difference normalisations correspond to the established global gamma and local gamma methods and the latter two normalisations allow for comparisons to be made with respect to the radiation oncology dose tolerances (QUANTEC) and planning constraints (RO specified) of each different contoured organ.

The values chosen from the QUANTEC dose tolerances were based on a conservative principle, where the values corresponding to the lowest toxicity were chosen if multiple dose tolerances were listed [25]. The values from the RO specified dose tolerances were provided to the dosimetrist for the specific patient, reflecting departmental protocols for specific treatment sites and/or the perceived achievable dose constraints for the patient. All dose tolerance values are specified to the maximum dose point (dmax). The QUANTEC dose tolerances and the range of RO specified dose tolerances for each structure in this study are listed in Table 2. The dose tolerance values for normalisation are assigned to the structures by creating a look-up table in Python where various names of the same structure in each plan are matched to a standardised naming. Gamma pass rates were calculated for all structures in all 78 treatment plans, with results from planning structures such as *ring* and *couch* ignored. Structure-specific gamma maps including selected OAR structures were plotted along with patient CT datasets to visualise the locations of the passing or failing regions with regards to the surrounding structures.

**Table 1:** The number of VMAT plans included in this study, including number of structures analysed per plan and the ranges of prescription dose and dose per fraction

Treatment sites	Number of plans	Number of structures analysed per plan	Number of structures analysed	Prescription dose (Gy)	Dose per fraction (Gy)
Brain	20	15	300	40–60	1.8–2.67
Head and neck	19	14	266	25–70	2–2.75
Thorax	20	5	100	30–66	1.5–3
Pelvis	19	5	95	45–78	1.8–2.24

**Table 2:** QUANTEC and the range of RO specified dose tolerances for the structures included in all 78 plans in this study. (Each plan only has a subset of the structures listed here) The dose tolerance values are specified to dmax

Structure	QUANTEC dose tolerances (Gy)	RO specified constraints (Gy)
BLADDER	65	35–65
BRAINSTEM	54	15–56
COCHLEA	45	5–45
FEMORAL HEAD	50	20–50
HEART	26	4–25
LARYNX	44	12–35
LENS	10	3–8
LUNG	7	10–20
MANDIBLE	50	50–73.5
OESOPHAGUS	34	25–34
OPTIC NERVE	55	30–54
ORAL CAVITY	30	10–35
PAROTID	20	10–35
PHARYNX	50	40–60
RECTUM	50	40–50
SMALL BOWEL	45	40–50
SPINAL CORD	50	15–50

## Results

Of all 78 plans, a total of 761 structures have been analysed, among which 357 structures have been specified an RO dose tolerance and also been contoured by the RO. Of the 357 structures, as listed in Table 3, global gamma has the highest average gamma pass rate and local gamma has the lowest average gamma pass rate. The local gamma pass rates also have the highest variation, resulting in some standard deviations exceeded the difference between the mean and maximum allowed gamma pass rates. QUANTEC and RO normalised gamma behave closer to global gamma but differs depending on structural dose tolerances. In all four treatment sites, 72.5% of structures in local gamma show poorer agreement than global gamma evaluation, whereas for QUANTEC normalised gamma and RO dose normalised gamma the proportions of structures showing poorer agreement than global gamma are 39.8% and 47.6% respectively.

**Fig. 2:**
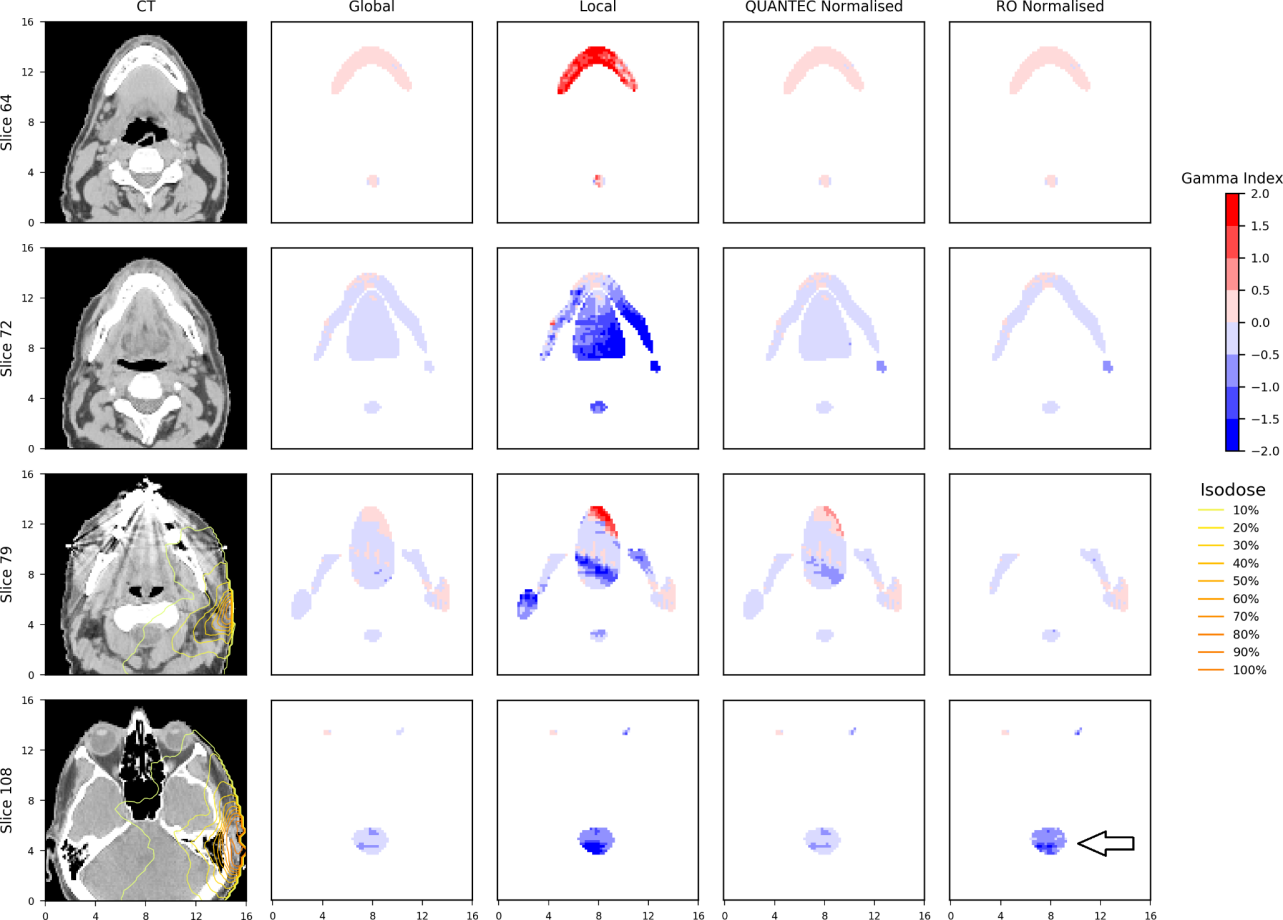
Gamma map of four selected slices from the 3D gamma analysis of a head-and-neck plan between MC and TPS calculated doses. The OARs shown in the figure are: oral cavity, L&R parotid, L&R cochlea, L&R optic nerves, L&R lenses, brainstem and mandible. Red indicates MC dose higher than TPS and blue indicates MC dose lower than TPS. Lighter colours indicate better agreement

**Fig. 3:**
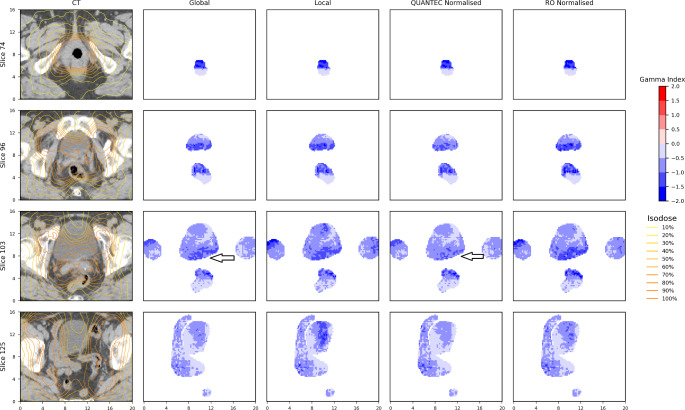
Gamma map of four selected slices from the 3D gamma analysis of a pelvis plan between MC and TPS calculated doses. The OARs shown in the picture are: femoral heads, bladder, rectum and small bowel

Two plans were chosen as examples as some OARs in these plans have highlighted the differences of behaviour between QUANTEC/RO normalised gamma and global gamma evaluation. Table 4 lists the percentage gamma pass rate calculated for the OARs from the two selected plans, using four different implementations of the gamma method. The corresponding gamma map of the two examples are shown in Figs. 2 and 3.

**Table 3:** Summary of structural gamma evaluation results, indicating the average gamma pass rates and standard deviation in parentheses, for all structures with RO dose tolerance in all 78 plans

		Global gamma evaluation	Local gamma evaluation		QUANTEC normalised gamma evaluation		RO dose normalised gamma evaluation	
Treatment site	Number of structures with an RO specified dose tolerance analysed	Average gamma pass rate (σ)	Average gamma pass rate (σ)	Number (%) of structures having lower gamma pass rate than global gamma	Average gamma pass rate (σ)	Number (%) of structures having lower gamma pass rate than global gamma	Average gamma pass rate (σ)	Number (%) of structures having lower gamma pass rate than global gamma
**Brain**	118	100% (0)	91.3% (14.1%)	36 (30.5%)	98.9% (3.0%)	6 (5.1%)	98.6% (3.1%)	7 (5.9%)
**Head and neck**	103	99.7 (0.5%)	82.0% (21.0%)	88 (85.4%)	97.6% (4.4%)	47 (45.6%)	96.8% (5.6%)	54 (52.4%)
**Thorax**	67	99.9% (0.2%)	86.3% (6.8%)	67 (100%)	97.6% (1.7%)	45 (67.2%)	99.0% (1.5%)	44 (65.7%)
**Pelvis**	69	97.2% (3.2%)	87.9% (7.6%)	68 (98.6%)	96.3% (3.8%)	44 (63.8%)	93.1% (6.4%)	65 (94.2%)
**Total /Average**	357	99.2% (1.9%)	86.9% (13.7%)	259 (72.5%)	97.6% (3.4%)	142 (39.8%)	96.9% (5.0%)	170 (47.6%)

**Table 4:** Examples of % gamma pass rate for structures where different implementations of the structural gamma method produced different results. The prescription doses were used for PTV RO dose tolerances in order to calculate RO normalised gamma for PTVs.

Treatment target	OAR / PTV	Global gamma pass rate (2%,2 mm)	Local gamma pass rate (2%,2 mm)	QUANTEC normalised structural gamma pass rate (2%,2 mm)	RO normalised structural gamma pass rate (2%,2 mm)
Left ear	Brainstem	100	63.0	100	96.9
	Left lens	100	90.0	100	90.0
	Right lens	100	100	100	100
	Spinal cord	100	70.2	100	100
	Left Cochlea	100	100	100	100
	Left parotid	100	96.5	98.0	99.5
	Mandible	100	80.9	100	100
	PTV	97.7	96.7	NA	97.0
Pelvis	Bladder	90.4	77.0	95.5	83.7
	Rectum	78.0	74.8	76.2	72.2
	Small bowel	89.1	85.7	85.3	85.3
	Left femoral head	99.4	90.4	99.0	94.3
	Right femoral head	97.0	84.4	96.1	88.8
	PTV	63.6	59.5	NA	59.2

Figure 2 is an example of the gamma map extracted from the 3D gamma analysis results between the MC and TPS calculated doses of a head-and-neck treatment plan. Four transverse slices through the three-dimensional datasets were extracted, representing the locations of some surrounding OARs. In this figure, each row shows a CT slice and the corresponding gamma map at that location in the treatment volume. The first column is from the CT dataset and all other columns represent the gamma map from the gamma evaluation with different normalisation values, indicated on the top of each column. As per legend on the right, the colours blue and red represent poor gamma agreement, with blue indicating evaluated dataset (MC doses) lower than reference dataset (TPS doses) and red indicating vice versa. Lighter colours indicate better agreement.

In this head-and-neck example, as shown from Fig. 2, global gamma comparison suggests strong agreement between the TPS and MC dose calculations, whereas the local gamma comparison is much more sensitive to differences between the two dose calculations. The QUANTEC normalised gamma behaves similarly to the global gamma overall. The RO normalised gamma effectively highlights the clinically relevant dose differences. As shown on slice 108, some regions of the brainstem (indicated by the arrow) had worse agreement on the RO normalised gamma than global and QUANTEC normalised gamma. This was because the RO has specified stricter dose tolerance for brainstem (20 Gy) than the QUANTEC recommendations (54 Gy) in this case. This failed region has highlighted that the dose difference to the brainstem may be of concern based on the RO-specified dose tolerance. However, since this dose difference is shown as being “cold”, which means MC calculated doses (evaluated doses) are lower than TPS calculated doses (reference doses). In this case underdosing OAR is probably not a concern to the clinician. The RO normalised gamma will not be performed if the RO has not specified dose tolerance for any particular structure, for example no oral cavity dose has been specified by RO in this case, as indicated by slice 79.

Figure 3 shows another example of the gamma comparisons between the MC and TPS calculated doses from a pelvis plan. On the four slices of the CT datasets extracted, the surrounding OARs shown are: femoral heads, bladder, rectum and small bowel. It is worth mentioning that on slice 103, the QUANTEC normalised gamma has better agreement than global gamma on the bladder (indicated by the arrows). This was because the QUANTEC bladder tolerance (dmax 65 Gy, standard fractionation) is greater than the prescription dose (50.4 Gy, 28 fractions) in this case, which would have been used for the global gamma normalisation.

## Discussion

In this study, we have performed structural gamma evaluation using four different types of normalisation between MC and TPS calculated doses for 78 retrospective patient plans of four different treatment sites. The structural gamma plot offers the benefits of retaining geometrical information while being able to provide quantitative results of disagreement of all structures in a nutshell. The structural gamma method is an objective assessment of comparing any two dose distributions. It generates quantitative results of normalised gamma values and pass rates for all contoured structures. The 3D gamma map provides an interactive view of the gamma results along with patient CT dataset that allows operators to scroll through CT slices and view or plot the gamma map of selected slices.

As we know the global gamma normalises to the maximum dose without considering dose tolerances of any structures. This can result in radiosensitive structures being overlooked and contribute to the lack of correlation with common dose-volume metrics demonstrated by Baran et al [6]. When the structural gamma method is used with reference to the global maximum dose the same fallibilities of the conventional global gamma method are reproduced (see elevated gamma pass rates for global gamma in Table 4), although opting to uniformly apply the global maximum allows analyses similar to the volume gamma work of Cozzolino et al. and Yi et al [13–15] to be completed.

When using the structural gamma method, analysis of dose differences as percentages of dose at each local point in the relevant part of the distribution similarly reproduces the known sensitivity of the local gamma method. Using structural gamma with this local normalisation is the most sensitive amongst the four normalisation methods, however it seems over-sensitive to small dose differences especially for structures with relatively high dose tolerances that may be less of a concern. This study identified several cases where the local gamma failed but the global and QUANTEC normalised gamma both passed (see Table 4). Overall, as shown in Table 3, the average local gamma pass rates in all four treatment sites are much lower than the QUANTEC/RO normalised gamma. This could indicate that the dose differences identified were not clinically significant considering the structure dose tolerances. According to Table 3, Local gamma evaluation results also have the highest variation between different plans, which is not desirable.

TG-218 [2] explicitly mentioned when determining critical structure dose tolerances, the dose difference criterion would ideally be customised for each organ. They stated that using customised organ dose tolerances would allow physicists to detect clinically relevant errors. Gamma passing metrics calculated for distributions as a whole are not necessarily correlated with clinically relevant dose differences in specific organs [6]. We believe the key advantage of the structural gamma method is to allow analysis to be performed with respect to RO specified OAR dose constraints. The structural gamma can perform analysis in selected structures according to the contours outlined from the patient CT dataset. These structures are usually surrounding OARs that the ROs are most concerned about. Compared to QUANTEC dose tolerance values, the RO specified OAR dose constraints are generally lower and more specific to each individual patient, as they are the dose limits the ROs specified with respect to their clinical experience and understanding of TPS capabilities. Therefore, the RO normalised structural gamma should be more clinically relevant than the QUANTEC normalised gamma which is based on more generic organ dose tolerances. It was found in some cases (such as Fig. 2, also see Table 4) that the QUANTEC normalised structural gamma evaluation passed but the RO normalised structural gamma evaluation failed (all being “cold”). These were generally found in structures where the RO had specified a much lower dose tolerance than the QUANTEC recommended values, based on knowledge of the patient characteristics (comorbidities, retreatments, etc.) and expected achievable OAR sparing. These example scenarios indicate that when structural gamma is used with reference to RO specified dose constraints, this method can produce more clinically relevant dose comparisons, taking into account organ specific and patient specific dose tolerances.

This structural gamma work has been limited by a focus on the dose difference aspect of the gamma evaluation. Dose tolerances were limited to absolute doses. A potential extension of this work would be to use EQD2 dose tolerances for the RO normalised gamma calculations. Another potential extension includes the use of structure-specific criteria that include geometric tolerances. For example, for stereotactic treatment plans where gradient indices are important, structure-specific DTA tolerances could also be nominated for certain structures. DTA tolerances may also be important for treatments planned on moving targets (e.g. lung, liver) with internal target volumes (ITVs) where there might be specific DTA criteria on some structures. Structural DTA tolerances can be specified and accounted for in the structural gamma evaluations, as an alternative or augmentation of the planning risk volume (PRV) concept. There are also some limitations due to the use of an in-house MC system which generally predicts lower doses than the clinical TPS. However, we believe it is sufficient in the current study as a tool for generating dose distributions for comparisons. In the future, if accessible, we believe using a clinical independent dose calculation tool would make our structural gamma tool a more clinically useful QA test.

To our best knowledge, no study has investigated dose comparison techniques in conjunction with variable specified structure dose tolerances. This method has the potential to be implemented clinically for comparisons of secondary dose calculations or PSQA results, supplementing the current method of performing global gamma analysis alone. This method nominally allows efficient identification of clinically relevant dose differences, which could reduce time spent on QA, hence improving QA efficiency.

## Conclusion

A method was developed to perform normalised gamma evaluation based on structure dose tolerances. This is an independent in-house developed method that has utilised open-source code based on an open-source programming language. This method has been tested on 78 retrospective VMAT patient plans between MC and TPS calculated doses and found to have captured clinically important errors that would have been missed by a standard global gamma evaluation. Due to the implementation of clinical dose tolerance values, especially RO specified dose constraints, the structural gamma method can be considered more clinically relevant than the widely used global gamma method or even the more sensitive local gamma method. The structural gamma method has the potential to be implemented clinically as a secondary check of PSQA, as a method of comparing two TPSs during TPS commissioning, or generally comparing any two dose distributions.
